# New record of the rare genus *Crinalium* Crow (Oscillatoriales, Cyanobacteria) from sand dunes of the Baltic Sea, Germany: epitypification and emendation of *Crinalium magnum* Fritsch et John based on an integrative approach

**DOI:** 10.11646/phytotaxa.400.3.4

**Published:** 2019-04-05

**Authors:** Tatiana Mikhailyuk, Oksana Vinogradova, Andreas Holzinger, Karin Glaser, Elena Samolov, Ulf Karsten

**Affiliations:** 1 M.G. Kholodny Institute of Botany, National Academy of Sciences of Ukraine, Tereschenkivska Str. 2, Kyiv 01004, Ukraine; 2 Department of Botany, Functional Plant Biology, University of Innsbruck, Sternwartestrasse 15, A-6020, Innsbruck, Austria; 3 University of Rostock, Institute of Biological Sciences, Department of Applied Ecology and Phycology, Albert-Einstein-Strasse 3, Rostock, D-18057, Germany

**Keywords:** *Crinalium magnum*, epitypification, Gomontiellaceae, integrative approach, mucilaginous sheath, 16S rRNA, 16S-23S ITS, phylogeny, TEM

## Abstract

Representatives of the Gomontiellaceae (Oscillatoriales) are rare and hence unstudied cyanobacteria with unusual morphology, distributed in terrestrial and aquatic habitats all over the world. Investigation of the group based on an integrative approach is only beginning, and to understand the actual biodiversity and ecology, a greater number of cultivated strains is necessary. However, some ecological traits of these cyanobacteria (e.g. low population densities, the absence of conspicuous growth in nature) led to methodological difficulties during isolation in culture. One species in the family Gomontiellaceae, *Crinalium magnum* Fritsch et John, is characterized by prominent wide and flattened trichomes, and represented by the non-authentic strain SAG 34.87. Detailed previous investigation of this strain clearly showed its morphological discrepancy with the original description of *C. magnum* and the genus *Crinalium* in general. The new isolate from maritime sand dunes of the Baltic Sea coast (Germany), however, revealed morphological characters completely corresponding with the diagnosis of *C. magnum*. Phylogenetic analysis based on 16S rRNA sequences indicated a position of the new strain inside Gomontiellaceae. Both morphology and ultrastructure of the strain are congruous with characters of the family. Epitypification and emendation of *C. magnum* are proposed since the ecology and habitat of the original strain are congruent with the type locality of this rare species (sand, Irish Sea coast, North Wales, UK). We expanded the description of *C. magnum* by details of the filament development and specified dimensional ranges for trichomes and cells, as well as by new data about the transversely striated structure of mucilaginous sheath.

## Introduction

The morphologically peculiar cyanobacterial genus *Crinalium* Crow is still poorly known. It belongs to the family Gomontiellaceae, with all the morphological features characteristic for its members: an unusual form of trichomes in the cross-section, very short cells, and a peculiar arrangement of thylakoids (Komárek & Anagnostidis 2005). Its main diacritical features are flattened trichomes, oval in cross-section. The genus (with type species *C. endophyticum* Crow) was described from mucilaginous colonies of *Aphanocapsa* Nägeli inhabiting wet rocks on a river-bank in North Wales (Crow 1927). Currently, *Crinalium* is represented by four species (Guiry & Guiry 2019), which are all free-living except the endogloeic *C. endophyticum*. *Crinalium glaciale* Broady et Kibblewhite (1991) was recorded in cryoconite pools on Antarctic glaciers. Two species described from biological soil crusts covering costal sand dunes include *C. magnum* Fritsch et John (1942) from the Irish Sea coast in Great Britain and *C. epipsammum* Winder, Stal et Mur (1990) from the Northern Sea coast in the Netherlands. The latter is the only representative of the genus, which was comprehensively described based not only on morphological, but also on ultrastructural, biochemical and genetic data (Winder *et al.* 1990).

In spite of its characteristic appearance and a rather large dimension for a cyanobacterium, *Crinalium* is a rarely occurring genus. Documented records of *C. endophyticum, C. epipsammum* and *C. glaciale* mainly relate to their type localities. Recent records of *C. endophyticum* in periphyton of highly polluted river in Israel (Barinova *et al.* 2004) and epiedaphic colonies on a margin of a drying pond in India (Jaiswal 2017) seem doubtful. Findings of *C. magnum* are more diverse in terms of ecological range and geographical distribution, but also quite infrequent. Information about its occurrence in modern compendiums and electronic databases is surprisingly incomplete. For example, in both editions of the “Freshwater algal flora of the British Isles” (Whitton *et al.* 2003: 61; John *et al.* 2011: 81) the genus *Crinalium* is only represented by *C. endophyticum* and, unconfirmed, *C. epipsammum* (“Several samples from the east coast of England and Scotland appear to be similar but detailed studies are needed.”). For inexplicable reasons, *C. magnum* was not included in the flora, although this taxon was described from the vicinity of Llandudno in North Wales (John 1942), which is 20 miles away from the type locality of *C. endophyticum* (Crow 1927). In the “Süsswasserflora von Mitteleuropa” *C. magnum* was mistakenly related to a “species outside Europe” as “known from India and Argentina” (Komárek & Anagnostidis 2005: 569).

In AlgaeBase (Guiry & Guiry 2019) information about the biogeographical distribution of *C. magnum* is incomplete. Type locality of this species is not given. In the section “Detailed distribution with sources”, the following is indicated: “Europe” (from Winder *et al.* 1990, which is a mistake since the paper describes *C. epipsammum*), South America: Argentina (Tell 1985), Brazil (De-Lamonica-Freire & Heckman 1996), South West Asia: India (Gupta 2012)”.

The description of *C. magnum* was based on the observations from a soil culture (Fritsch & John 1942), but the type material was not preserved. The strain SAG 34.87 is the only known cultured representative of this species. However, according to Bohunická *et al.* (2015) a comprehensive analysis of members of the family Gomontiellaceae, including information of cell dimensions, filament length and circular shape in cross-section testify that the strain SAG 34.87 does not correspond to either the original diagnosis of *C. magnum* (Fritsch & John 1942) or to the genus *Crinalium* in general (see below). It could be possible that the strain SAG 34.87 belongs to *Hormoscilla* Anagnostidis et Komárek species rather than *Crinalium*.

During the investigation of biological soils crusts from sand dunes of the Baltic Sea coast (Germany) that yield several interesting taxa (Mikhailyuk *et al*. 2019), a cyanobacterium was discovered that was characterized by wide, flattened, ribbon-like trichomes and very short cells, which morphologically corresponding to *Crinalium magnum*. This new strain was isolated in culture and conducted a complex investigation, including the morphology of trichomes, the ultrastructure of cells, and molecular-phylogenetic analysis. The obtained material allowed us to improve the description of the species and to conduct its epitypification. Since the type species was unavailable for genetic analysis, the strain Hg-6-6 was selected as the epitype supported by molecular phylogenetic data.

## Materials and methods

### Strain isolation and culture conditions

The strain mentioned in the study was isolated from the samples of biological soil crusts collected from the coastal sand dunes in Heiligendamm (Mecklenburg-Vorpommern, Germany, GPS data: 54.14486164 N, 11.8534391 E). The material was collected 25.10.2013; the strain was isolated in December 2013. The crust was dominated by *Klebsormidium crenulatum* (Kützing) Lokhorst. Fragments of the crust (approximately 6 × 6 cm in size) were collected by cutting the soil crust and transferred into a Petri dish; the samples were air-dried and stored in the dark.

A small amount of crust was placed in Petri dishes with Bold Basal agarized (1.5%) medium (1N BBM; Bischoff & Bold 1963). Cultures were grown under fluorescent light (25 μmol photons • m^-2^ • s^-1^) with a 12:12 light: dark photoperiod at +20 ± 5 °C An unicyanobacterial culture was obtained using the stereomicroscope Olympus ZS40 (Tokyo, Japan), and purified from other organisms by multiple transfers. The purified strain was maintained under the same conditions on agarized (1.5%) BG-11 medium (SigmaAldrich; Stanier *et al.* 1971). Additionally purification of the strain from fungal contamination was performed using fungicide cycloheximide (100 mg/L of BG-11 medium) with following transferring on pure agarized BG-11 medium.

The isolated culture (Hg-6-6) is kept in the algal culture collection at the University of Rostock, Germany; the strain was also deposited in SAG, University of Göttingen, Germany (SAG 2581) and culture collection of the M.G. Kholodny Institute of Botany of the National Academy of Sciences of Ukraine (IBASU-A-693). From the culture, a herbarium specimens were prepared—a young (3 weeks old) culture was preserved in 2% and 4% formaldehyde, in a 15 ml glass bottles. Preserved material was then deposited in the Algotheca of the M.G. Kholodny Institute of Botany of the National Academy of Sciences of Ukraine (AKW-32501).

### Light and transmission electron microscopy

Morphological examinations started with 2 week-old cultures and continued during the 6 months of cultivation; old cultures (aged 8 months and more) were also studied to provide details on the development of the filaments. The study was performed using an Olympus BX51 light microscope with Nomarski DIC optics. Photomicrographs of living material were taken with digital camera Olympus UC30 attached to the microscope, and processed by software cellSens Entry. Average values of the dimensional measurements (x_a_) were calculated using Microsoft Excel. Mucilage was stained with both drawing ink and an aqueous solution of methylene blue.

Samples were fixed for transmission electron microscopy (TEM) using a standard chemical fixation protocol (2.5% glutaraldehyde, 1% OsO_4_ in 10 mM caccodylate buffer, pH=6.8) according to Holzinger *et al.* (2009). Samples were dehydrated in increasing ethanol concentrations, transferred to modified Spurr’s resin and heat polymerized. For TEM ultrathin sections were prepared, counterstained with uranyl acetate and Reynold’s lead citrate, and investigated in Zeiss LIBRA 120 transmission electron microscope at 80 kV. Images were captured with a TRS 2k SSCCD camera and further processed using Adobe Photoshop software (Adobe Systems Inc., San José, California, USA).

### DNA isolation, PCR, sequencing and phylogenetic analysis, secondary structure of RNA

DNA of the cyanobacterial strain was extracted using the DNeasy Plant Mini Kit (Qiagen GmbH, Hilden, Germany) according to the manufacturer’s instructions. Nucleotide sequences of the 16S rRNA gene together with 16S-23S ITS region were amplified using Taq PCR Mastermix Kit (Qiagen GmbH), and primers SSU-4-forw and ptLSU C-D-rev (Marin *et al.* 2005) in a thermocycler Tgradient Thermoblock (Biometra, Germany) under the conditions described in a previous paper (Mikhailyuk *et al.* 2016). PCR products were cleaned using a Qiagen PCR purification kit (Qiagen GmbH) according to the manufacturer’s instructions. Cleaned PCR products were sequenced commercially by Qiagen Company using primers SSU-4-forw, Wil 6, Wil 12, Wil 14, Wil 5, Wil 9, Wil 16, and ptLSU C-D-rev (Wilmotte *et al.* 1993; Marin *et al.* 2005). The resulting sequence (16S rRNA gene, partial sequence; 16S-23S ITS region, complete sequence; and 23S rRNA gene, partial sequence) was assembled and edited using Geneious software (version 8.1.8; Biomatters); it was deposited in GenBank under the accession number MK211234.

In order to find the closest relative to our strain, nucleotide sequences of the Synechococcales and Oscillatoriales representatives available in GenBank, as well as the BLASTn queries (http://blast.ncbi.nlm.nih.gov) were used. Multiple alignment of the 16S rRNA gene nucleotide sequences were made using MAFFT web server (version 7, Katoh & Standley 2013), followed by the manual editing in program BioEdit (version 7.2). The evolutionary model that fitted best to the used dataset was selected based on the lowest AIC value (Akaike 1974) calculated in MEGA (version 6, Tamura *et al.* 2013).

The phylogenetic tree based on the Bayesian inference analysis was computed in MrBayes 3.2.2 (Ronquist & Huelsenbeck 2003) using an evolutionary model GTR + G + I with 5,000,000 generations. For the Bayesian analysis two runs of the four Markov Chain Monte Carlo were made simultaneously, with the trees taken every 500 generations. Split frequencies between runs were below 0.01 at the end of calculations. The trees selected before the likelihood rate reached saturation were subsequently rejected. The tree topology was verified by the maximum likelihood analysis (ML) made with the program GARLI 2.1., and the neighbor-joining analysis (NJ) based on sequence differences with uniform rates in 1,000 bootstrap replications, calculated with MEGA (version 6, Tamura *et al.* 2013). Genetic distances inside the family Gomontiellaceae were calculated in the program MEGA using uniform rates.

Models of the secondary structure of 16S-23S ITS region of the cultured strains of *Crinalium* and *Hormoscilla* were built according to the model proposed in Hašler *et al*. 2014. Helices were folded with the online software Mfold (Zuker 2003) and visualized in the online tool PseudoViewer (Byun & Han 2009).

## Results

### Molecular phylogeny and secondary structure of 16S-23S ITS region

**Figure 1:**
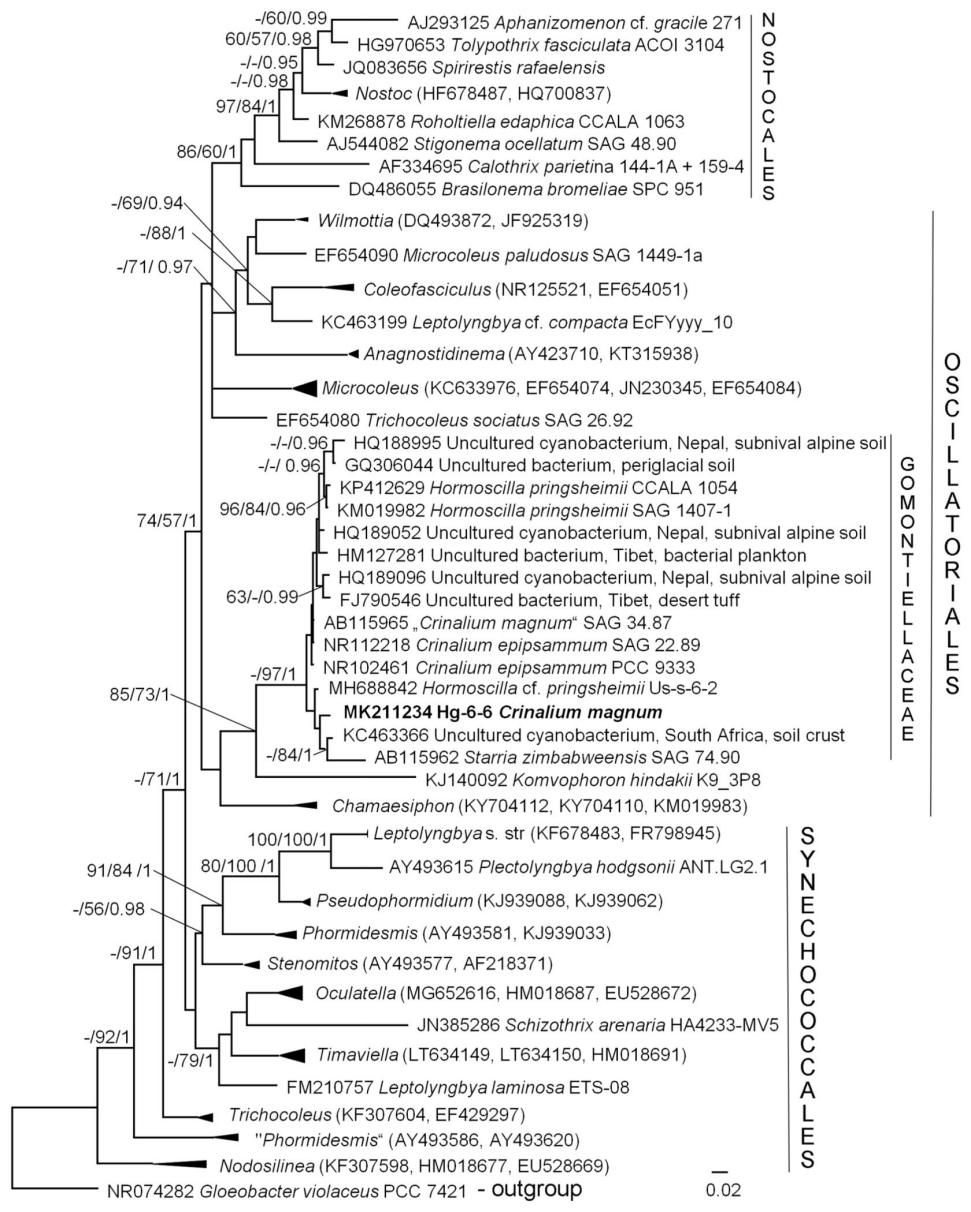
Molecular phylogeny of Synechococcales and Oscillatoriales based on 16S rRNA sequence comparisons. A phylogenetic tree was inferred by the Bayesian method with Bayesian Posterior Probabilities (PP), Maximum Likelihood and Neighbor Joining bootstrap support (BP). From left to right, support values correspond to Neighbor Joining, Maximum Likelihood BP and Bayesian PP; BP values lower than 50% and PP lower than 0.8 not shown. Strain in bold represents newly sequenced cyanobacteria. Clade designations follow Osorio-Santos *et al.* (2014) and Bohunická *et al.* (2015).

Phylogenetic analysis based on 16S rRNA sequence comparison showed that the investigated original strain Hg-6-6 joined the clade of the family Gomontiellaceae (Fig. 1). All members of this clade including several environmental sequences showed close similarity and formed a separate highly supported lineage within Oscillatoriales. The isolate Hg-6-6 represented a separate lineage within Gomontiellaceae, among other strains of the genera *Hormoscilla* and *Crinalium*. Pairwise comparison of 16S rRNA gene sequences of different Gomontiellaceae strains, *Hormoscilla*, *Crinalium*, and *Starria* Lang, as well as outgroup taxon *Komvophoron hindakii* Hašler et Poulíčková, showed a close similarity of representatives of the family (Table 1). The identity of nucleotides of 16S rRNA gene of the two related genera strains *Hormoscilla* and *Crinalium* varied from 99.0 to 100%.

**Figure 2:**
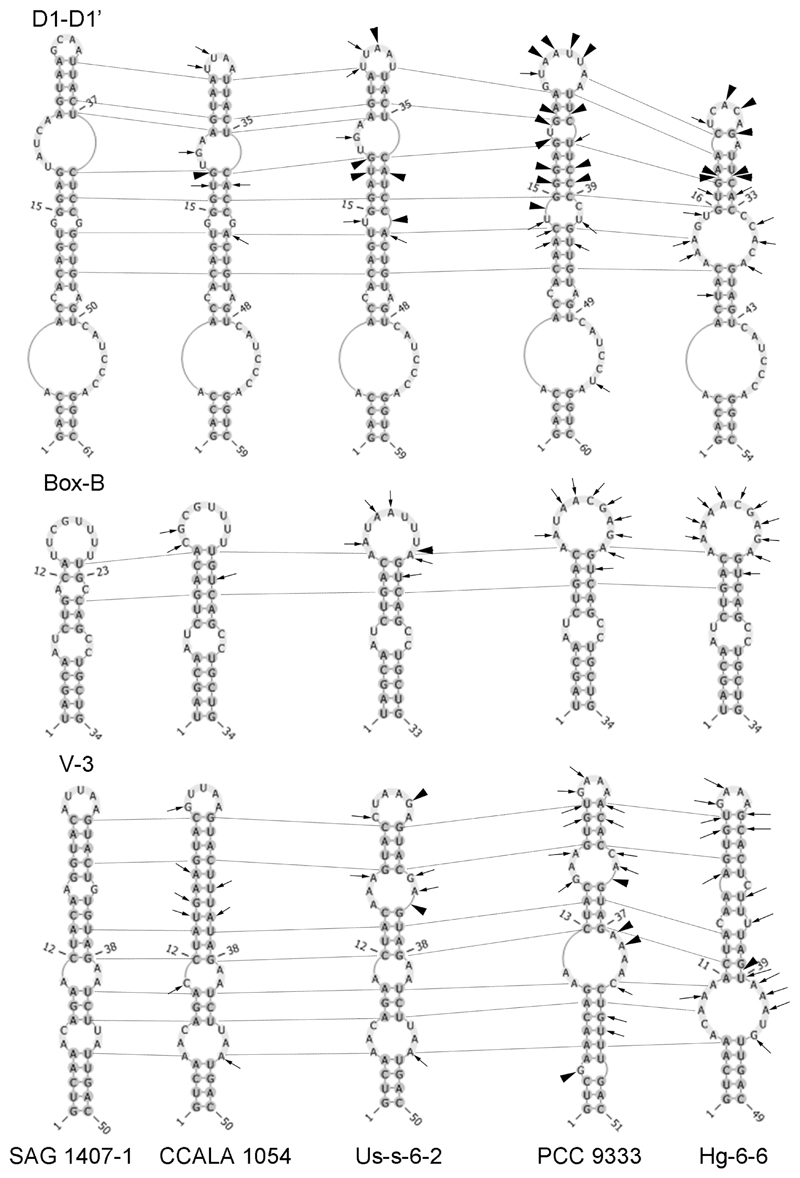
Secondary structure of the main informative helices of region 16S-23S ITS of cultured strains of *Hormoscilla* and *Crinalium*. All differences between strains are presented in comparison with authentic strain of *H. pringsheimii* (SAG 1407-1). Variable bases are shown with arrows, places of insertions/deletions of base pairs are marked with arrowheads, homological base pairs among different strains are indicated with dotted lines.

Secondary structures of the main informative helices (D1-D1’, Box-B and V-3) of region 16S-23S ITS of newly sequenced strain Hg-6-6 and publically available sequences of strains *Hormoscilla pringsheimii* (SAG 1407-1 (KJ140105), CCALA 1054 (KP412629) and Us-s-6-2 (MH688842)) and *Crinalium epipsammum* (PCC 9333(CP003620)) showed general similarity especially prominent among *Hormoscilla* strains (Fig. 2). Helices of *Crinalium* strains were characterized by increase of internal (V-3) and terminal loops (Box-B) and elongation/shortening of the upper part of helix D1-D1’ due to numerous insertions or deletions of base pairs.

### Morphological observations

Thick filaments in a colorless sheath were found in an enrichment culture of the sand dune biological soil crust. The cyanobacteria formed bright blue-green spots, several millimeters in diameter on the agar surface. Preliminarily identified as a species of *Lyngbya* Agardh ex Gomont, closer examination revealed the flattened shape of the trichomes, oval in cross-section. Therefore, this isolate was identified as a representative of the genus *Crinalium*. Comparison of morphological features of our strain with known taxa of this genus showed close similarity to *C. magnum* (see below). Morphological characters of the investigated strain, in comparison to the other species of the genus, were summarized in Table 2.

**Figure 3:**
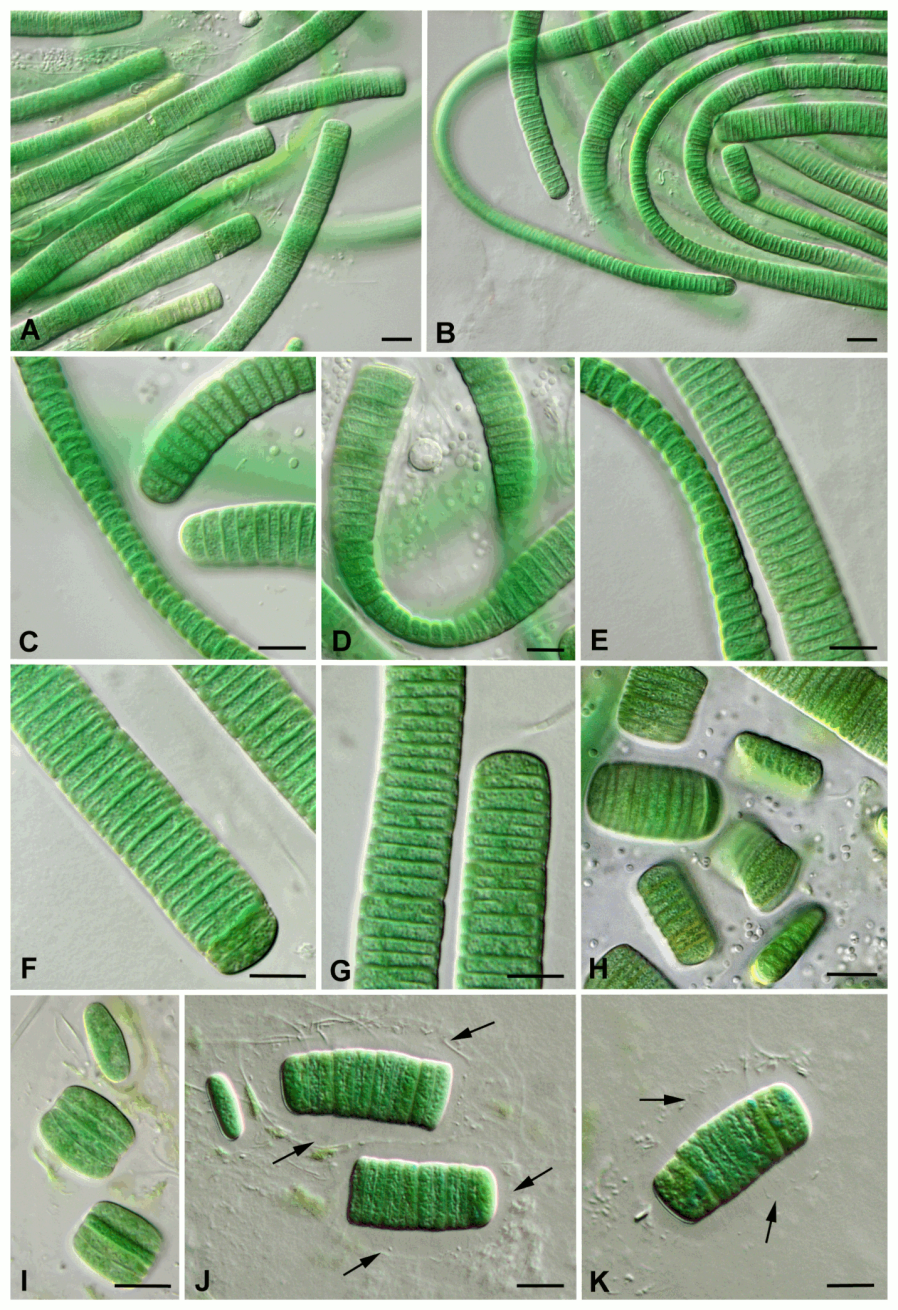
Light micrographs showing an overview of living filaments of *Crinalium magnum* strain Hg-6-6. A, B. Irregular clusters with trichomes varying in length. C–E. Trichomes lying in two planes. F, G. Details of trichomes and terminal cells with a thickened outer margin. H–K. Fragmentation of trichomes in old cultures (6 and more months). Arrows mark the sheath. Scale bars: 10 μm.

Due to the isolation of the C. *magnum* strain Hg-6-6, the possibility to study the characteristics of the development of filaments and the details of cell morphology was given. Single filaments, developing on the surface of the agar, gradually formed irregular clusters and bright blue-green colonies with a smooth surface. Depending on the age, the length of the trichomes varied from several tens to hundreds of microns (Figs 3 A, B, H–K). In young cultures they were relatively long; with aging trichomes disintegrated into either fragments containing a small number of cells or even single cells. Filaments were straight or bent, strap-shaped (Figs 3 C–E), and oval in cross-section (Fig. 3 I). Trichomes were bright blue-green, flattened from the sides, slightly constricted at the non-granulated cell walls, not getting narrow at the ends, 5–7 μm thick (x_a_ = 6.37μm), (9.5) 10–16 (18) μm wide (x_a_= 13.76). Cells were always shorter than the trichome width, 2–4 μm long (x_a_ = 2.86 μm). Terminal cells were rounded, sometimes with slightly thickened cell wall (Figs 3 F, G).

**Figure 4:**
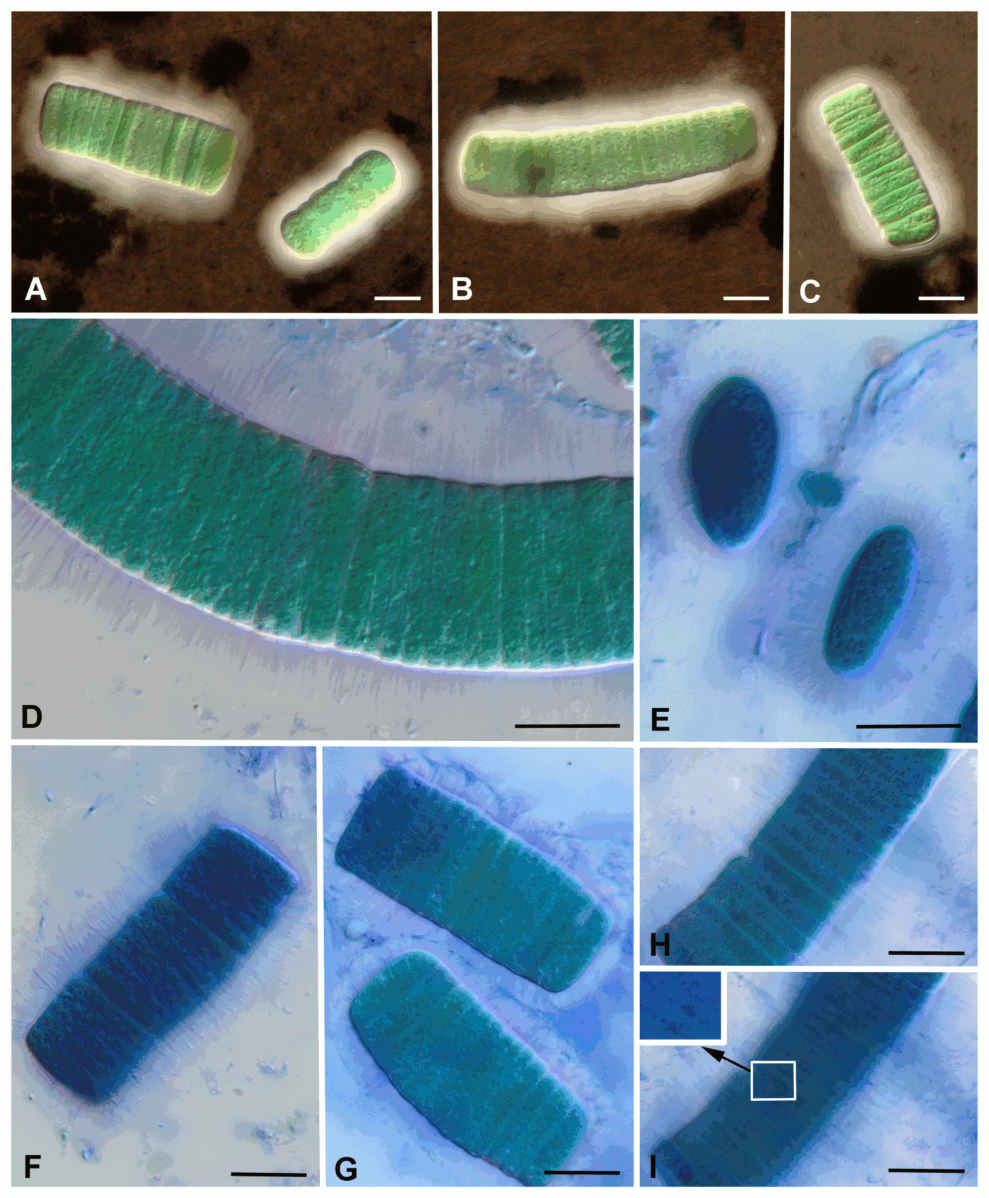
Staining of mucilage envelope of *Crinalium magnum* Hg-6-6. A–C. Staining with drawing ink showed difluent mucilage envelope. D–I. Staining with methylene blue showed striated structure of mucilage. E. Separate cells in lateral position with mucous microfibriles radiated from the cell wall. H, I Trichome in optical section (H) and in surface view (I) with increased portion showed mucous microfibrils arranged by rows along cross cell walls. Scale bars: 10 μm

Mucilaginous sheath appeared colorless, poorly visible without staining (Figs 3 J, K). After staining with drawing ink it became apparent as 5–8 μm thick envelope, with uneven but distinct margins (Figs 4 A–C). Staining with an aqueous solution of methylene blue showed a distinctive structure: exopolysaccharide (EPS) layer around the trichomes formed wide, transversely striated envelope (Figs 4 D–I). The EPS envelope completely covered the trichomes, and its striped appearance was not due to the folded surface of the sheath. Mucous microfibrils radiated from the cell wall, perpendicular to the trichome plane (Fig. 4 E), and were arranged in rows closely associated with the cross cell walls (Fig. 4 I).

### Ultrastructure

**Figure 5:**
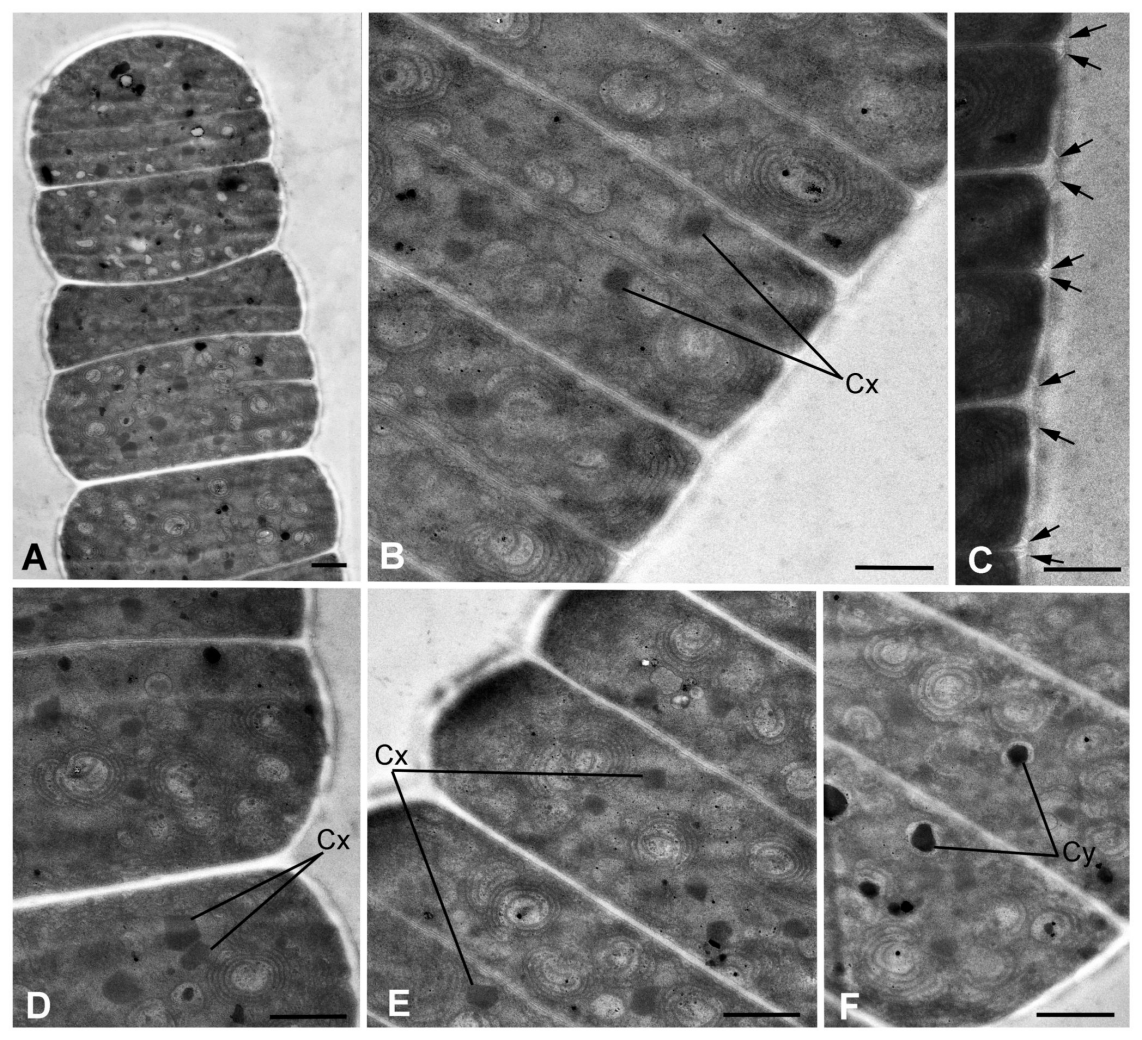
Ultrastructure of *C. magnum* strain Hg-6-6. A. Longitudinal section of the trichome showing its general organization. B, D–F. Portions of filaments showing typical arrangement of helically twisted, swirl-like thylakoids and cell inclusions. C. Junctional pores (arrows) closely associated with the cross walls. Cx, carboxysomes; Cy, cyanophycin granules. Scale bars: 1 μm

TEM of the strain Hg-6-6 revealed thylakoids arranged both peripheral and parallel to the cell wall, organized as helically twisted tangles in the cytoplasm (Figs 5 A–F). The cell wall had an uneven surface and contained rows of junctional pores. They were located circumferentially and closely associated with the cross walls from both sides (Fig. 5 C). Inclusions observed in the cells were identified as carboxysomes and cyanophycine granules (Figs 5 B, D–F).

## Discussion

### Placement of *Crinalium magnum* in the molecular phylogeny of Gomontiellaceae

Molecular phylogenetic analyses based on the 16S rRNA gene revealed close relationship of the strain Hg-6-6 to the other representatives of the family Gomontiellaceae. It once again confirmed the monophyly of this family, with the genus *Komvophoron* as a sister group and its placement inside Oscillatoriales (Hašler *et al.* 2014; Bohunická *et al.* 2015, see Fig. 1). Recently it was also found that representatives of the Gomontiellaceae are closely related with *Chamaesiphon* Braun (Kurmayer *et al*. 2017) that was also shown on our phylogenetic tree (see Fig. 1).

However, the position of *Crinalium* strains on the phylogenetic tree raises a question of relationship between the genera (namely *Crinalium* and *Hormoscilla*). In our research, the original strain of *C. magnum* Hg-6-6 forms a separate lineage and is nested among strains of both genera. Bohunická *et al.* (2015) also noted a high molecular similarity of the mentioned genera despite clear morphological differences (flattened versus non-flattened trichomes). The pair-wise nucleotide identity of the 16S rRNA gene of *Crinalium* strains SAG 34.87, SAG 22.89 and Us-s-6-2, and *Hormoscilla* strains SAG 1407-1 and CCALA 1054 was 99%. Strains SAG 34.87 and SAG 22.89, representing “*Crinalium magnum*” and *C. epipsammum,* showed 100% similarity (Bohunická *et al.* 2015; Table S3). Despite the fact that calculated levels of pairwise similarity are much higher than that required for the separation of genera (Stackebrandt & Goebel 1994), the authors came to the conclusion that clear morphological and ecological differences between *Hormoscilla* and *Crinalium* “provide a solid basis for retaining their taxonomic resolution in the framework of a polyphasic approach” (Bohunická *et al.* 2015: 1045). The inclusion of Hg-6-6 here confirmed that the strains of both genera are closely related (see Table 1). Therefore, generic borders inside Gomontiellaceae are still unclear and perhaps generic concept should be clarified in future with more strains included in the analysis. On the other hand perhaps 16S rRNA is not sufficient marker for some closely related cyanobacterial taxa, therefore other genetic markers should be used in order to resolve relationships within Gomontiellaceae.

For better resolution secondary structures of the main informative helices of 16S-23S ITS region of *Hormoscilla* and *Crinalium* were used (see Fig. 2). These structures are also characterized by similarity. Although some differences are visible among representatives of two genera, *Hormoscilla* and *Crinalium*, due to numerous insertions/deletions of base pairs in the last genus. Unfortunately secondary structure of RNA of authentic strain of *Crinalium epipsammum* (SAG 22.89) was not analyzed since 16S-23S ITS fragment is not publically available. Therefore perhaps analysis of secondary structure of 16S-23S ITS region of all available strains of Gomontiellaceae will give more information for better understanding generic borders of two genera.

Hg-6-6 is genetically (and morphologically, see below) distant from the strain SAG 34.87, which was previously identified as *C. magnum* (see Fig. 1). Some doubts concerning morphological and genetic data regarding the strain SAG 34.87 were already expressed in Bohunická *et al.* (2015) and discussed below.

### Morphology and ultrastructure of *Crinalium magnum*

The morphological characters of strain Hg-6-6 completely correspond with the diagnosis of *C. magnum* (Fritsch & John 1942; see Table 2). They share the same cell dimensions, characteristics of the filaments (both short and long, ensheathed, sheath closed at the apices) and trichomes (not narrowed at the ends, slightly constricted at the ungranulated cell walls, terminal cells with slightly thickened outer margin). Morphology of the strain SAG 34.87 previously identified as *C. magnum* essentially differs from the species description, in terms of the cell size and shape, and morphologically resembles a *Hormoscilla* species (Bohunická *et al.* 2015). However, genetically this strain is close to *Crinalium epipsammum,* which contradicts the morphological data (e.g. non-flattened trichomes) (see Fig. 1, Bohunická *et al.* 2015). Anyway, strain SAG 34.87 is genetically distant from Hg-6-6, hence it represents another taxon which is not related (morphologically or genetically) to *Crinalium magnum*.

Morphological observations of *C. magnum* Hg-6-6 supplemented the description of this species by details of the filament development and refined dimensional ranges for the trichomes and cells (see Table 2). The present study provides new information concerning the EPS envelope, which is also interesting in the context of the genus as a whole: the presence of the sheath in *Crinalium* was doubted since the publication of Crow (1927). Indeed, the protologue described very briefly: “Trichomes … vaginate, sheath thin…” (Crow 1927, p. 165). However, in the cited paper one can find additional details concerning the sheath: “…each filament consists in the adult state of trichome and sheath … the sheath is thin but clearly defined, membranous, slightly brownish… the thickness of the sheath being less than 1 μm” (Crow 1927: 161–162). Unfortunately, the quality of the figure with a “…portion of filament with trichome broken, showing sheath” (Crow 1927: 164, Fig. 2B) is extremely poor. This was the reason both Geitler (1932: 981) and Elenkin (1949: 1847) doubted the presence of a sheath in *Crinalium.* The description of *Crinalium* given in “Süsswasserflora” (Komárek & Anagnostidis, 2005: 568) also states: “Sheaths lacking, or form very fine and diffluent gelatinous layer enveloping the trichome”. At the same time, in the description of *C. magnum* var. *colloncurense* Guarrera (also cited in *Süsswasserflora*) filaments in mucilaginous envelopes which formed mucous masses on rocks are described (“Filamentos… unidos por contacto de las vainas individuales formando masas gelatinosas…” (Guarrera *et al.*, 1995: 290).

Actually, three from the four known species of the genus have ensheathed filaments (see Table 2). In protologues of *C. magnum* and *C. glaciale* it was noted that the sheath was visible only after staining. In both cases, drawing ink was used for the visualization of sheath. In this study we observed the presence of EPS layer around trichomes (similar to the ones shown in Fig. 8 (A) in Fritsch & John 1942) even without staining. As seen from the micrographs, the sheath of filaments both unstained (Figs 3 J, K) and stained with drawing ink (Figs 4 A–C) look homogenous. Staining with methylene blue revealed a thick and transversely striated sheath in *C. magnum* (Figs 4 D–I). It is known that sheath with microfibrils radiating from the cell wall surface is typical for immotile trichomes (Komárek & Anagnostidis 2005: 27). The sheath structure of *C. magnum* strain Hg-6-6, and the fact that no signs of motility in the material studied here suggests immotility of this species. However, Fritsch & John (1942: 393) claimed the opposite: “the isolated threads being usually buried in the sand of the moist culture”; so further study is necessary.

TEM of the strain Hg-6-6 revealed ultrastructural pattern typical for representatives of the family Gomontiellaceae, mostly because of specific helically twisted, swirl-like thylakoids (Komárek *et al.* 2014; Bohunická *et al.* 2015). We also found junctional pores closely associated with the cross walls, their organization into multiple parallel rows is a unique feature of the family (Bohunická *et al.* 2015). TEM micrographs from this article (Bohunická *et al.* 2015, Figs 2 e, f) also showed EPS excretion from the cells of *Hormoscilla pringsheimii* Anagnostidis et Komárek to some extent similar to the striated sheath of *C. magnum* Hg-6-6 observed by us on LM level (see Figs 4 D–I). As can be seen from these figures, mucous microfibrils radiate from the cell wall perpendicularly to trichome plane, and they are arranged in rows closely associated with cell cross walls (see Fig. 4 I). Thus, it is possible to assume that junctional pores may be responsible for the EPS excretion and formation of the striated sheath. Function of junctional pores is still a disputable question (Bohunická *et al.* 2015). Observations via light microscopy of the secretion process in two species of Oscillatoriales and Nostocales provided direct evidence that the junctional pore complex is the actual site of mucilage secretion (Hoiczyk & Baumeister 1998). As was recently shown using TEM phylogenetically related to Gomontiellaceae species of *Chamaesiphon* are also characterized by presence of numerous pores in cell wall excreting mucilage (Kurmayer *et al.* 2017).

### Ecology and distribution of *Crinalium magnum*

Initially, *C. magnum* was characterized as a species confined to sandy soils (John 1942); the following findings strongly expanded its ecological range. *C. magnum* was cited from India where it was recorded in red soil (Mitra 1951), rice fields (Sanyal *et al.* 2012), thermal springs (Pathan 2014), stagnant water (Jaiswal 2017), and even in the soil of cyanide damps where it occurred as an abundantly growing taxon (Karthikeyan 2016). This species was also found in the phytoplankton of a shallow lake in Argentina (Guarrera *et al.* 1972) and in a rain pool in Brazil (Heckman 1998). In Antarctica, *C. magnum* was reported from mineral soils and growing epiphytically on mosses of the Victoria Land (Broady 1986). At first glance, these data show a species with wide ranges of both ecological tolerance and geographical distribution. Unfortunately, most findings of *C. magnum* in the mentioned papers are limited to its name and did not provide any descriptions, figures or molecular data, therefore it is impossible to assess their reliability.

Our record of this species fully complies with the type locality of *C. magnum* characterizing it as an inhabitant of epipsammon. Our finding is quite close to the type locality, ecologically and geographically since *C. magnum* was described from marine coastal sand dunes in North Wales, UK (John 1942).

The introduction of molecular analyzes in the practice of studies of cyanobacterial diversity will help to accurately identify the limits of ecological tolerance of this species. Recent molecular investigation of Gomontiellaceae showed that different taxa of the group are quite common in environmental samples from terrestrial habitats all over the world (Bohunická *et al.* 2015). On the other hand, they are characterized by low population densities and usually do not produce high biomass in nature, therefore they are frequently overlooked on a morphological level. The same reasons cause technical difficulties during the isolation of these species and the establishment of cultures. Moreover, cultivated strains of the genera *Crinalium* and *Hormoscilla* are quite sensitive and slow-growing, which makes Gomontiellaceae rare and challenging cyanobacteria to study.

### Proposed taxonomic treatment

*Crinalium magnum* Fritsch et John 1942, *Annals of Botany,* New Series 6 (23): 371–395, Fig. 8 (A), Emend. O.M. Vynogr. et Mikhailyuk (Figs 3–5)

*Emended diagnosis*: Filaments solitary or in irregular clusters, straight or curved, band-shaped. Sheath hyaline, colorless, poorly visible without staining, thick, closed at the apex, with an uneven edge, 5–8 μm wide, transversely striated due to mucous microfibrils radiating from the cell wall perpendicular to trichome plane (visible after staining with methylene blue). Trichomes bright blue-green, flattened from the sides, oval in cross-section, vary in length from 20–40 μm up to 300–500 μm, (9.5) 10–16 (18) μm wide, 5–7 μm thick, not attenuated at the ends, slightly constricted at the cross-walls. Cells always shorter than wide, 1/4–1/9 times as long as wide, 2–4 μm long (x _a_ = 2.86 μm). Apical cells rounded, sometimes with a slightly thickened cell wall. Thylakoids partly arranged parietally also form helically twisted tangles in the whole cytoplasm.

*Type locality*: in firm sand, with or without a certain amount of humus; sand dunes on the coast of the Irish Sea near Llandudno, North Wales, UK.

*Lectotype* (designated here): Fig. 8 (A) in Fritsch et John 1942.

*Epitype* (designated here)*:* AKW-32501, culture material of epitype strain Hg-6-6 (IBASU-A-693) preserved in 4% and 2% formaldehyde, Algotheca of the M.G. Kholodny Institute of Botany of the National Academy of Sciences of Ukraine.

*Epitype strain*: Hg-6-6 was deposited in SAG, University of Göttingen, Germany, under number SAG 2581 and in the Culture Collection of the M.G. Kholodny Institute of Botany of the National Academy of Sciences of Ukraine under number IBASU-A-693.

*Comments*: The epitype strain completely corresponds to the diagnosis of *Crinalium magnum* (Fritsch & John 1942). The species diagnosis was supplemented by the details of filament development and refined dimensional ranges for trichomes and cells, as well as new information concerning mucilaginous sheath structure. The epitype strain was isolated from biological soil crusts on maritime sand dunes, coast of the Baltic Sea, Heiligendamm, Mecklenburg-Vorpommern, Germany. This habitat is ecologically very similar to the type locality and is part of the same geographical region (Europe).

## Figures and Tables

**Table 1:** Percent identity of the 16S rRNA gene (1053 positions) of the cultured representatives of the family Gomontiellaceae in comparison with the closest relative.

	Taxa, strains	1	2	3	4	5	6	7	8
1	*Crinalium magnum* Hg-6-6								
2	“*Crinalium magnum*” SAG 34.87	99.3						
3	*Crinalium epipsammum* SAG 22.89	99.3	100					
4	*Crinalium epipsammum* PCC 9333	99.2	99.9	99.9				
5	*Hormoscilla pringsheimii* SAG 1407-1	99.3	99.2	99.2	99.1			
6	*Hormoscilla pringsheimii* CCALA 1054	99.1	99.1	99.1	99.0	99.9		
7	*Hormoscilla* cf. *pringsheimii* Us-s-6-2	99.2	99.7	99.7	99.6	99.1	99.0	
8	*Starria zimbabweensis* SAG 74.90	96.4	96.6	96.6	96.5	96.7	96.7	96.7
9	*Komvophoron hindakii* clone K9 3P8	91.1	91.5	91.5	91.5	91	90.8	91.2	90.5

**Table 2:** Comparison of morphological features of the strain Hg-6-6 with species of the genus *Crinalium* Crow based on their protologues (a dash indicates the absence of data).

Character	*C. endophyticum* Crow 1927	*C. epipsammum* Winder, Stal et Mur 1990	*C. glaciale* Broady et Kibblewhite1991	*C. magnum* Fritch et John 1942	*C. magnum* var. *colloncurense* in Guarrera *et al*. 1995	*C. magnum* Hg-6-6
Filaments	doubled by means of a bend about their middle, often coiled in a lax spiral, have the form of a hairpin	straight or irregularly wavy	single, not aggregating to form a visible plant mass, straight, never hooked	straight, never spirally twisted or bent	thick, 13–13.5 μm wide, single or connected by mucous envelopes, forming gelatinous masses of uncertain morphology	single or in irregular clusters, straight or bent
Sheath	thin but clearly defined, membranous, slightly brownish	absent	completely surrounding the trichomes including the apices, homogeneous, colourless, up to 4 μm thick	narrow, diffluent, closed at the ends and visible only after staining	Present	colorless, 5–8 μm thick, after staining looks transversely striated
Trichome length	40–250 μm	varies with culture conditions, 200–400 (1000) μm	10–200 μm, mostly 40–110 μm	reachs a length of 0.5 mm, although most of them are shorter	–	varies from several tens to hundreds of microns, starts to be shorter with aging of culture
Trichome thickness	–	2–2.5 μm	7.5–9 μm	–	–	5–7 μm
Attenuation to the ends	slightly attenuated	not attenuated	not attenuated	not attenuated	–	not attenuated
Constrictions	not constricted	slightly constricted	distinctly constricted	slightly constricted	slightly constricted	slightly constricted
Cell width	2–3(4) μm	5–7 μm	13–26 μm, mostly 18–22 μm	upto to 18 μm	12 μm	13–18 μm
Cell length	–	1.0–1.5 μm	1.5–3.5 μm	1.5–3 μm	2.5–4.5	2–4 μm
End cells	conical-rounded	not different in morphology from intercalary cells	rounded to broadly conical, usually slightly longer than intercalary cells, up to 5 μm long	slightly longer than the others, with a convex and slightly thickened outer margin	convex and longer, with or without thickening on the outer wall	rounded, sometimes with slightly thickened cell wall
Occurrence	endogloeic in mucilage *Aphanocapsa fonticola* Hansgirg colonies, the damp face of a rock in Fairy Glen, Betws-y-Coed, N Wales, GB	surface layer of sandy soil of coastal dunes of the Northern Sea, the Netherlands	amongst sediments in cryoconite ponds on glaciers in southern Victoria Land, Antarctica	on the surface of the seaside sand-dunes, coast of the Irish Sea, North Wales, UK	epilithic, Kollon Kura River, Argentina	in biological soil crusts on the sand dunes, coast of the Baltic Sea, Heiligendamm, Mecklenburg-Vorpommern, Germany
